# Microbial and Geochemical Diversity of Laguna Timone, an Extreme Hypersaline Crater Lake in Patagonia (52° S)

**DOI:** 10.3390/microorganisms13081957

**Published:** 2025-08-21

**Authors:** Carolina Henríquez, José M. Pérez-Donoso, Nicolás Bruna, Mauricio Calderón, Leonardo Fadel Cury, Paulo Quezada, Gustavo Athayde, Poldie Oyarzún, Anelize Bahniuk

**Affiliations:** 1 LAMIR Institute, Graduate Program in Geology, Universidade Federal do Paraná, Curitiba 81531-980, Paraná, Brazil; cury@ufpr.br (L.F.C.); paulo.quezada@ufpr.br (P.Q.); anelize.bahniuk@ufpr.br (A.B.); 2 BioNanotechnology and Microbiology Laboratory, Center for Bioinformatics and Integrative Biology (CBIB), Facultad de Ciencias de la Vida, Universidad Andres Bello, Santiago 8320000, Chile; n.brunarivera@gmail.com; 3 Centro C+, Facultad de Ingeniería, Universidad del Desarrollo, Santiago 7610658, Chile; mccaldera@gmail.com; 4 Hydrogeological Research Laboratory (LPH), Graduate Program in Geology, Universidade Federal do Paraná, Curitiba 81530-000, Paraná, Brazil; gustavo.athayde@ufpr.br; 5 Laboratorio de Análisis de Sólidos, Universidad Andrés Bello, Santiago 8320000, Chile; poldie.oyarzun@unab.cl

**Keywords:** microbial communities, microbial mats, hypersaline lake, geochemistry, southern Patagonia

## Abstract

Extreme environments, such as hypersaline habitats, hot springs, deep-sea hydrothermal vents, glaciers, and permafrost, provide diverse ecological niches for studying microbial evolution. However, knowledge of microbial communities in extreme environments at high southern latitudes remains limited, aside from Antarctica. Laguna Timone is a hypersaline crater lake located in a Pleistocene maar of the Pali Aike Volcanic Field, southern Patagonia; the lake was formed during basaltic eruptions in a periglacial setting. Here, we report the first integrative characterization of microbial communities from biofilms and microbial mats in this lake using high-throughput 16S rRNA and ITS gene sequencing, along with mineralogical and hydrochemical analyses of water, sediments, and carbonates. Bacterial communities were dominated by the genera *Enterobacterales* ASV1, *Pseudomonas*, *Oscillatoria*, *Nodularia*, and *Belliella*, with site-specific assemblages. Fungal communities included *Laetinaevia*, *Ilyonectria*, *Thelebolus*, *Plectosphaerella*, and *Acrostalagmus*, each showing distinct distribution patterns. These baseline data contribute to understanding microbial dynamics in hypersaline maar environments and support future investigations. This integrative approach highlights key microbe–mineral relationships and underscores the potential of Laguna Timone as a natural laboratory for exploring biosignature formation and microbial adaptation in chemically extreme environments, both on early Earth and potentially beyond.

## 1. Introduction

Microbial mats were the first terrestrial ecosystems in early life on Earth, paving the way for the evolution of complex ecosystems [1,2,3]. The naturally hypersaline environments where they developed are considered one of the probable settings for the emergence of life on Earth [4], and places where some microorganisms currently inhabiting the environment might be descendants of primordial life forms [5]. This hypothesis is related to the balance between sodium and potassium ions within cells, which stimulates the concentration of prebiotic molecules due to evaporation, and increases the possibility that vacuoles and phosphate membranes developed as a strategy to survive extreme salinity [6]. Therefore, the halophilic organisms within their environments make these sites perfect candidates for studies on the origin of life, and the evolution of such organisms [5].

The limited number of examples of such environments includes a few hypersaline lakes, such as Hamelin Pool and Lake Clifton in Western Australia [7] and Storr’s Lake and Highborne Cays in the Bahamas [8,9,10], as well as the Great Salt Lake Desert in Utah [11], the Atacama Desert [12], and the Argentine Puna [13,14,15,16]. In general, the hallmarks of these lakes include high concentrations of carbonate and/or salts, high salinity, and a high pH (9.0–12.0) [17,18,19]. A new example of such an environment is the hypersaline and alkaline crater lake Laguna Timone, placed within an extinct Pleistocene maar of the Pali Aike Volcanic Field in southernmost South America. In this area, microbial mats withstand extreme environmental conditions, including high salinity, elevated pH levels (~10), low temperatures reaching −8 °C, intense solar radiation, strong winds, and a semi-arid climate. Although the presence of biofilms and microbial mats has been documented [20], detailed studies are lacking.

The crater lake Laguna Timone was formed during phreatomagmatic eruptions of basaltic magmas in Middle Pleistocene times once the ice caps formed during the Great Patagonian Glaciation started to retreat [21]. The origin of the Middle Pleistocene to Holocene transitional to alkaline-type basalts of the Pali Aike Volcanic Field is related to the heating and partial melting of deep lithospheric mantle in a subduction zone, induced by the up-rising of hotter sub-slab asthenosphere material through a slab window [22]. In a broad sense, the interactions of ice-sheet dynamics, volcano–tectonic events, and the prevailing sub-Antarctic climatic conditions generated the environmental conditions that presently support the colonization of an array of microorganisms in the crater lake. Therefore, the unusual extreme conditions and the still-unexplored microbial diversity, combined with minimal human interference, make this an exceptional location in which to document, for the first time, the microbial community composition.

In this study, we integrate a comprehensive suite of microbiological, mineralogical, and geochemical analyses of microbial mats, carbonates, sediments, and water. Our objective was to produce a detailed characterization of the microbiome present in an unexplored Quaternary hypersaline crater lake by employing high-throughput 16S rRNA gene sequencing. This approach enabled us to characterize the composition of the indigenous microbial communities inhabiting this extreme ecosystem. As this is one of the first descriptive studies of microbial diversity in hypersaline lakes in the southernmost regions of South America, the resulting data provide a valuable baseline for understanding microbial dynamics in these environments. This work lays the foundation for future investigations into the functional traits, ecological interactions, and evolutionary adaptations of extremophile communities in cold, saline volcanic systems. Moreover, it offers relevant analogs not only for reconstructing early Earth conditions, but also for assessing the potential for life in similarly extreme environments beyond our planet.

## 2. Geological Setting

**Figure 1:**
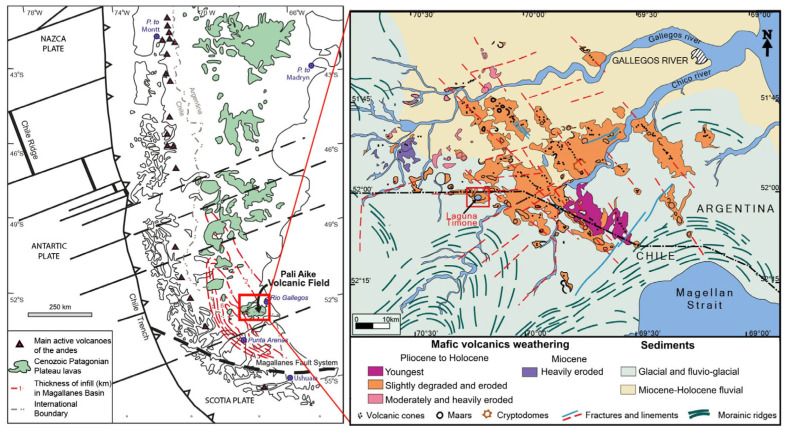
Regional map of Patagonia showing the distribution of basaltic rocks like the Pali Aike Volcanic Field. The inset shows the geological context of Laguna Timone (red square) in the Pali Aike Volcanic Field (source: adapted from [30,31,32]).

The Cenozoic geodynamic evolution of southernmost South America is mainly due to the interactions among the South American, Antarctic, and Scotia tectonic plates. The widespread basaltic magmatism of Pali Aike Volcanic Field (PAVF) is related to the opening of an asthenospheric window beneath the lithosphere of southern South America in response to the subduction of the Chile Ridge [23,24,25]. The late Miocene to Quaternary PAVF (3.8 to 0.17 Ma) extends over an area of approximately 4500 km^2^ in Argentina and Chile [26], emplaced on top of the Mesozoic to Cenozoic sedimentary succession of the Magallanes Basin [27,28] (Figure 1). The underlying sedimentary rocks consist of Oligocene marine sandstones and shales deposited during a marine transgression from the Atlantic Ocean, which in turn are overlain by up to 1 km thick molasse-type sediments related to the early Miocene tectonic uplift of the Andes. These sequences are covered by Pliocene to Pleistocene fluvioglacial deposits generated during glacial and interglacial cycles, including very extensive glacial advances [29].

Three main volcanic stages have been proposed for the generation of the alkaline basalts of the PAVF [33]. The first of them was the one that created the greatest volume of volcanism, which was directly associated with the passage of the asthenospheric window, forming extensive outcrops of plateau basalts. The remaining two events generated a variety of volcanism-related morphologies, such as spatter and slag cones, tuff rings, lava flows, and maars. The maars of the PAVF formed during phreatomagmatic eruptions caused by the interactions of mantle-derived basaltic magmas with groundwater, surface water, and/or permafrost in a proximal periglacial environment [34].

Laguna Timone is a site located at the lowest altitude of a group of maars and corresponds to a subcircular emission center surrounded by a tuff ring and scoriaceous basaltic deposits, to some extent with mantle xenoliths, with thicknesses of tens of meters. Laguna Timone is one of the few water-filled craters in the PAVF. Its filling is provided by groundwater, probably derived from the melting of ancient ice masses, with contributions from episodic rainwater and snowfall events. The hypersalinity of this type of water, usually referred to as brines, prompts the consideration that these fluids are of magmatic and/or hydrothermal origin. Laguna Timone does not have permanent tributaries or surficial outflow, and thus represents an endorheic lake with a restricted water supply. It is emplaced in a basaltic volcanic field and exposed to the influence of strong westerlies and low temperatures [20].

## 3. Climate Conditions

Climatic conditions in southern Patagonia are controlled by Antarctic ice masses and wind circulation regimes from the west [35]. A characteristic feature of the sub-Antarctic climate is the predominance and strong intensity of the (westerlies) that predominate during the year [36]. The Humboldt Current (Pacific coast) and the Malvinas/Falkland Current (Atlantic coast) transport cold water to the north along the South American continental margins, simultaneously reducing atmospheric heating [37]. Moreover, high solar radiation melts the extensive ice fields each summertime and promotes evaporative conditions at the surface [38]. The Andean Mountain range acts as a topographic barrier for humid winds, producing a rain shadow in the eastern slopes of the Andes, where PAVF is located. This results in an abrupt decrease in precipitation to less than 200 mm per year, with regular distribution throughout the four seasons, giving rise to a semi-arid and desert climate [29].

## 4. Methods

### 4.1. Sampling

**Figure 2:**
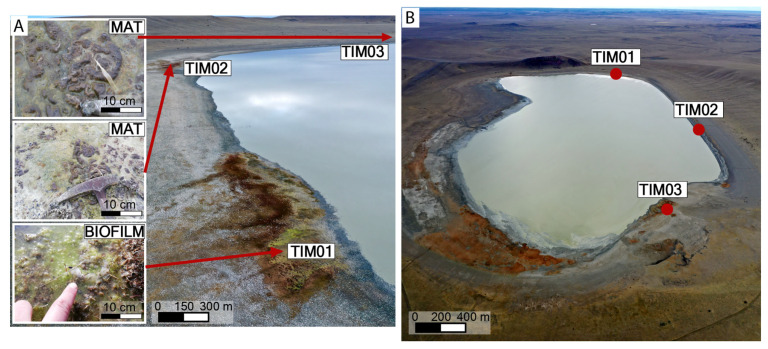
Location of the microbial samples around Laguna Timone. (**A**) The images show the locations of TIMO 1, TIMO 2, and TIMO 3 on the margin of the lake. (**B**) The drone image shows the spatial locations of the samples around the crater lake.

**Figure 3:**
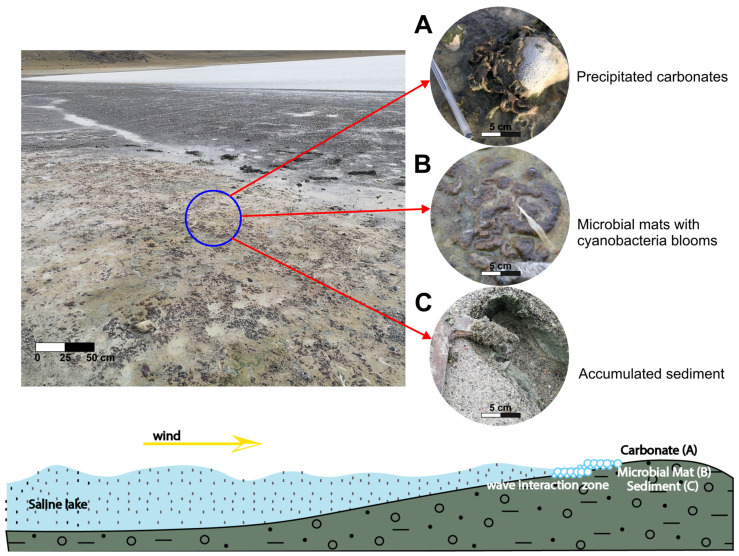
The images show microbial mats developed on sediments that are partially covered with carbonate precipitates on the margin of the crater lake, where the microbial mats are above the sediments and below the carbonate precipitated. The schematic model of Laguna Timone illustrates where the microbial system develops along the margin of crater lake. There is wave action zone that reworks the observed microbial mats, and prompts a mixture of all components of the system. The blue circle encompasses the locations of the images in the scheme representing the (**A**) carbonates, (**B**) microbial mats, and (**C**) sediment.

Three distinct samples were collected for this study: TIMO 1, representing biofilms, and TIMO 2 and TIMO 3, representing microbial mats (Figure 2). TIMO 1 was obtained from submerged surfaces at a depth of approximately 5 cm within a water-filled pool ad-jacent to the lagoon. The biofilm was green in color, exhibited a slimy texture, and contained visible gas bubbles. In contrast, TIMO 2 and TIMO 3 were collected from microbial mats located on the exposed sediment surface along the same shoreline, in direct contact with the lake water. Each sample covered an area of approximately 5 cm^2^. The mats were about 2 mm thick and brownish-green in color, and exhibited a homogeneous structure with no visible stratification. A distinctive morphological feature was their boomerang-like shape (Figure 3). The nature of this extreme area in Patagonia presents significant logistical and practical challenges that limit the possibility of obtaining biological replicates. Despite the lack of biological replicates, the three samples collected were carefully selected as representative of the microbial and mineralogical diversity of the region.

To minimize the risk of contamination, samples for DNA extraction were collected using gloves, sterile scalpels, and tweezers. These were stored in PowerSoil tubes (Qiagen, Hilden, Germany) at −20 °C and processed within one week for genomic analysis. Negative extraction and PCR controls were included throughout to monitor potential contamination during DNA extraction and amplification. Additional samples for scanning electron microscopy (SEM) and epifluorescence microscopy were placed in sterile bags and stored at 4 °C in the field.

These microbial mats, together with the associated biofilm, constitute the only microbial assemblages identified within the study area. Their respective sampling locations are indicated in Figure 3. Due to the extreme environmental conditions and the remoteness of the Patagonian site, significant logistical limitations prevented the collection of seasonal replicates. To address this, three representative samples were strategically selected to capture the microbial diversity of the region.

In addition to the microbial mat samples, complementary environmental materials were collected, including water, carbonate aggregates, and surface sediments spatially associated with the microbial communities. Fieldwork was conducted at the end of the austral winter, under the cold and arid conditions typical of the region. Water temperature, pH, electrical conductivity (EC, µS cm^−1^), and total dissolved solids (TDS, mg L^−1^) were measured in situ using a Horiba multiparameter probe (Horiba, Kyoto, Japan), while total alkalinity was determined with a PF-12Plus photometer. For chemical analysis, water samples were filtered through 0.45 μm surfactant-free cellulose acetate (SFCA) syringe filters; due to the high concentration of dissolved solids, filtration was repeated as needed. Filtered water for cation analysis was stored in acid-washed bottles and kept at 4 °C until processing. For isotopic analysis, water was sampled directly from the microbial mat areas and injected (0.2 to 1 mL) into pre-prepared 12 mL vials sealed under helium and containing phosphoric acid. Both the carbonate aggregates covering the mats and the surface sediment samples were collected in sterile polyethylene bags.

### 4.2. Microbial Mats and Biofilm Microscopy Characterization

The microbial mats were studied using an epifluorescence microscope (MF606 BW OPTICS, Nanjing, China) equipped with five wavebands in order to detect the natural fluorescence of microorganisms under ultraviolet (UV) radiation.

The microstructural characterization of the microbial mats and associated minerals was performed using a JEOL 6010LA scanning electron microscope (SEM) (JEOL Ltd., Tokyo, Japan) equipped with secondary electron, backscattered electron, and energy-dispersive spectroscopy (EDS; model EX123 94410T1L11) detectors (JEOL Ltd., Tokyo, Japan). The equipment was operated at 20 kV, with a magnification range of 5× to 300,000×. Thin surface layers (~1.5 mm thick) were manually subsampled from the microbial mats. To preserve the native microstructure, the samples were dehydrated using critical point drying (CPD). SEM imaging of biological material was also conducted using a Bal-tec CPD030 system (Bal-tec AG, Balzers, Liechtenstein).

High-resolution imaging, qualitative and quantitative chemical analyses, and structural characterization of the crystalline phases and materials present on the microbial mats were performed using a FEI TITAN G2 Transmission Electron Microscope (TEM) (Thermo Fisher Scientific, Hillsboro, OR, USA) operated at 300 kV. Samples were prepared by affixing particles onto a 200-mesh copper carbon film, which was subsequently metallized prior to analysis.

### 4.3. Analysis of Microbial Communities

DNA was extracted from the microbial mat and biofilm samples from Laguna Timone using the DNeasy^®^ PowerSoil^®^ Pro (QIAGEN, Hilden, Germany), following the manufacturer’s protocol, with 250 mg of soil per extraction. Total DNA concentration was quantified using a Qubit fluorometer (Invitrogen, Carlsbad, CA, USA). Sequencing was performed in the Argonne National Laboratories, using the Earth Microbiome Project barcoded primer set adapted for the Illumina HiSeq2000 and MiSeq platforms (Sequence data were processed using QIIME 1.9.1) [39,40].

The analyses were conducted in R v4.0.3 and RStudio v1.4.1717, using the DADA2 package v1.18 software package for paired-end fastq files (https://benjjneb.github.io/dada2/ (accessed on 22 May 2025)) [41]. For the analysis of the 16S rRNA gene, the V4 region was amplified using primers 515F (5′GTGCCAGCMGCCGCGGTAA3′) and 806R (5′GGACTACHVHHHTWTCTAAT3′). The SILVA database (version 138) was used for the taxonomic assignments [42]. For the ITS analysis, the forward primer 5′CTTGGTCATTTAGAGGAAGTAA3′ and the reverse primer 5′GCTGCGTTCTTCATCGATGC3′ were used to amplify the fungal ITS1 region. The UNITE database (version 9.0) was used for the taxonomic assignments [39]. Sequences were trimmed at 250 bp for forward reads and 200 bp for reverse reads, based on their respective quality profiles. The “phyloseq” object obtained from the taxonomic analysis excluded sequences with fewer than two reads. Raw sequences were deposited under NCBI SRA BioProject PRJNA1139992.

Alpha diversity was assessed using amplicon sequence variants (ASVs) to calculate diversity indices and richness estimators for the bacterial communities in the samples. The Chao1, Shannon, and Simpson indices were employed to measure species richness, abundance, and dominance. The variance-stabilizing transformation was applied to normalize data, using the DESeq2 v1.28.1 [43] R package.

### 4.4. Water Chemistry and ^13^C_DIC_ Composition

Water samples were filtered using fiberglass and cellulose ester membranes prior to analysis. Concentrations of ions were measured in mg L^−1^, and ion abundances were calculated in milliequivalents per liter (meq L^−1^). Chloride, sulfate, fluoride, phosphate, nitrite, nitrate, silica, iron, and manganese concentrations were determined by colorimetric methods using a UV-Vis spectrophotometer (MN^®^ UV/Vis II, Macherey-Nagel, Düren, Germany). Carbonate, bicarbonate, hydroxide, calcium, magnesium, and total hardness were measured by titration using a BRAND^®^ Titrette^®^ digital burette (CO KG, Wertheim, Germany). Sodium and potassium concentrations were deter-mined by flame emission photometry using a Celm FC 280 photometer (Celm, s.r.o., Prague, Czech Republic). Total dissolved solids were quantified by gravimetric analysis.

The carbon isotope composition of the dissolved inorganic carbon (δ^13^C_DIC_) was analyzed at the LAMIR Institute, using a Thermo^®^ GasBench II coupled with a Thermo^®^ (Thermo Fisher Scientific, Bremen, Germany) Delta V Advantage isotope ratio mass spectrometer (IRMS). The analysis was conducted in continuous flow mode, and results are expressed in delta notation (δ), in per mil (‰), relative to the international standard Vienna Pee Dee Belemnite (V-PDB).

### 4.5. Chemical and Mineralogical Analyses of Sediment and Carbonate

Bulk samples of sediment were analyzed by TESCAN-TIMA equipment (TESCAN, Brno, Czech Republic) for auto-mated mineralogical mapping. Image analysis was performed simultaneously with SEM backscatter electron images combined with X-ray fluorescence. The analytical technique is automated, and it utilizes a robust database that transforms EDS chemical data to mineralogy. The instrument is housed in Chile, at Solutions in Microscopy and Applied Mineralogy (SEMMA).

X-ray diffraction analysis of the carbonate samples was performed using a PANalytical Empyrean diffractometer (Malvern Panalytical, Almelo, The Netherlands) equipped with an X-Celerator detector and CuKα radiation. Measurements were taken at a scan rate of 0.5° per minute, with an operating voltage of 40 kV and a current of 30 mA. Carbonate classification was based on d-spacing values obtained from the XRD spectra, following the criteria described in [44].

### 4.6. Isotope Composition (δ^13^C and δ^18^O)

Isotopic compositions of δ^13^C and δ^18^O in the microbial mats and carbonate samples were determined using a Thermo Fisher Scientific Delta V Advantage mass spectrometer (Thermo Fisher Scientific, Bremen, Germany). The CO_2_ for spectrometry was generated in the laboratory by reacting carbonate powder samples with 100% phosphoric acid at 72 °C using the Gas Bench II preparation and introduction system. Isotopic ratios were calibrated against the international Vienna Pee Dee Belemnite (VPDB) standard. Data processing was performed using the Isodat 3.0 software.

## 5. Results

### 5.1. Microbial Mats and Biofilm Communities in Laguna Timone

**Figure 4:**
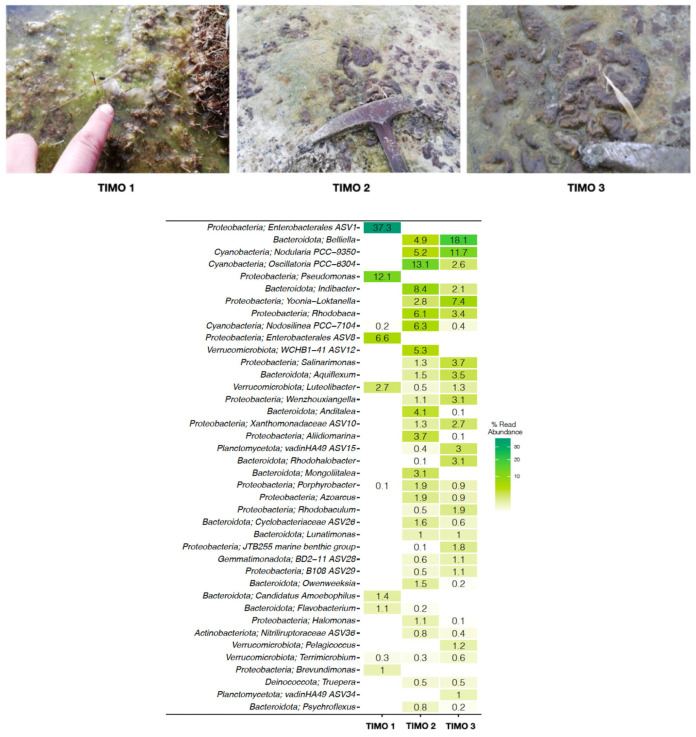
Relative abundance of bacterial genera in TIMO 1, TIMO 2, and TIMO 3, based on 16S rRNA gene sequencing. Dominant taxa included *Enterobacterales* ASV1 and *Pseudomonas* in TIMO 1, cyanobacteria such as *Oscillatoria* and *Nodularia* in TIMO 2, and *Belliella* in TIMO 3.

The genetic analysis revealed distinct taxonomic profiles across the three sampling sites (Figure 4). TIMO 1 was dominated by the phyla Proteobacteria and Bacteroidota, with *Enterobacterales* ASV1 (~37%) and *Pseudomonas* (~12%) as the most abundant genera. Other lower-abundance taxa included *Enterobacterales* ASV8 (~6%), *Luteolibacter* (~3%), *Candidatus Amoebophilus* (1.4%), *Flavobacterium* (1%), and *Brevundimonas* (1%). Other taxa, such as *Terrimicrobium*, *Nodosilinea*, and *Porphyrobacter*, were present at relative abundances below 1%. TIMO 2 was dominated by the phyla Bacteroidota, Proteobacyeria and Cyanobacteria, with *Oscillatoria* (~13%), *Indibacter* (~8%), *Nodosilinea* (6.3%), *Rhodobaca* (~6%), *WCHB1-41* ASV12 (~5%), *Nodularia* (~5.2%), and *Belliella* (~5%) as the most abundant genera. TIMO 3 was also dominated by the phyla Bacteroidota, Proteobacteria, and Cyanobacteria, with its dominant genera including *Belliella* (~18%), *Nodularia* (~11%), and *Loktanella* (~7%). Phylum- and family-level summaries have been included in the Supplementary Material (Figures S1 and S2).

**Figure 5:**
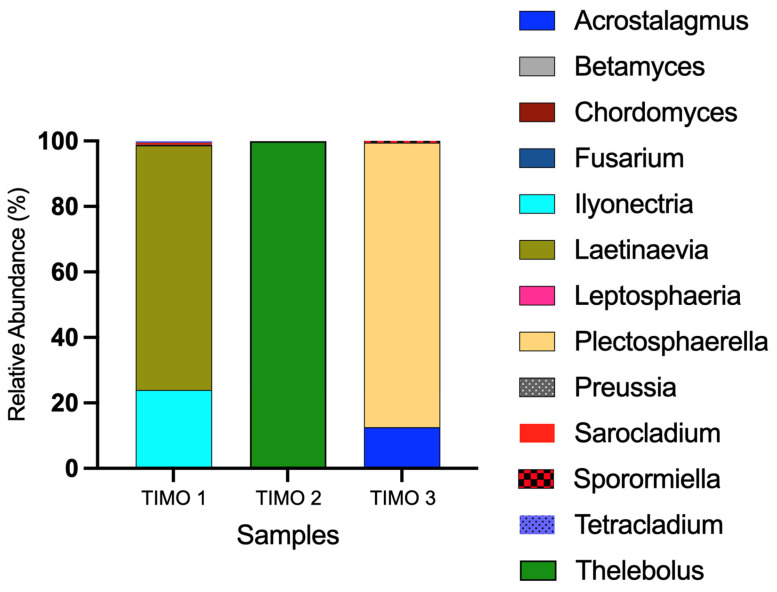
Genus-level fungal composition in the TIMO 1–3 samples based on ITS sequencing. TIMO 1 was dominated by *Laetinaevia* (~75%) and *Ilyonectria* (~24%), TIMO 2 by *Thelebolus* (~99.9%), and TIMO 3 by *Plectosphaerella* (~87%) and *Acrostalagmus* (~12.5%). Additional low-abundance genera were also detected at each site.

Fungal communities differed among the three sampling sites, based on the ITS data (Figure 5). Taxonomic profiles showed notable variation, with each site characterized by a distinct dominant genus. In TIMO 1, the most abundant genera were *Laetinaevia* (~75%) and *Ilyonectria* (~24%), with lesser representation of *Sarocladium*, *Preussia*, *Tetracladium*, *Sporormiella*, *Fusarium*, and *Chordomyces*. TIMO 2 was dominated almost exclusively by *Thelebolus* (~99.9%). In contrast, TIMO 3 showed a predominance of *Plectosphaerella* (~87%) and *Acrostalagmus* (~12.5%), in addition to low-abundance genera such as *Sporormiella* and *Leptosphaeria*. These results highlight compositional differences in fungal communities across the sites. Phylum- and family-level summaries have been included in the Supplementary Material (Figures S3 and S4).

**Figure 6:**
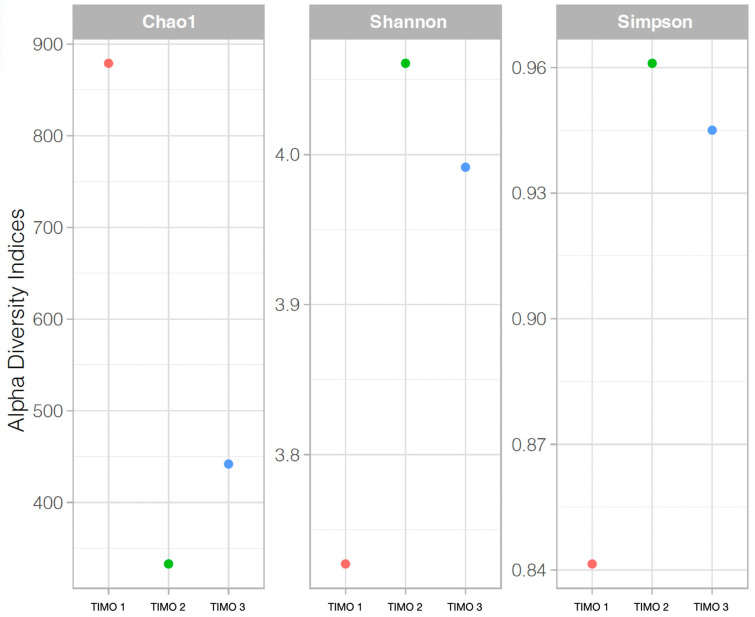
Alpha diversity indices (Chao1, Shannon, and Simpson) for microbial communities in TIMO 1 (biofilm), TIMO 2, and TIMO 3 (microbial mats). Chao1 reflects estimated species richness, Shannon combines richness and evenness, and Simpson measures dominance. TIMO 1 (red) showed the highest richness but lowest evenness (high dominance), while TIMO 2 (green) exhibited low richness but the highest evenness. TIMO 3 (blue) presented intermediate values for all three indices.

Alpha diversity indices indicated low overall diversity across the samples, with variations among the sites (Figure 6). TIMO 1 showed the highest richness, based on Chao1, while TIMO 2 exhibited the highest evenness, as reflected in the Simpson index. TIMO 3 displayed intermediate values for all three indices. Rarefaction curves for both the 16S and ITS datasets are provided in Supplementary Figures S5 and S6, confirming sufficient sequencing depth across samples.

### 5.2. Characterization of Microbial Mats

The TIMO 1 and TIMO 2 samples are not distinguishable based on their macroscopic and microscopic characteristics. Both are thinner than 5 mm and consist of an external EPS-rich layer (>1 mm thick) covering a greenish-brown layer approximately 2 mm thick. These microbial mats do not show any visible stratification.

**Figure 7:**
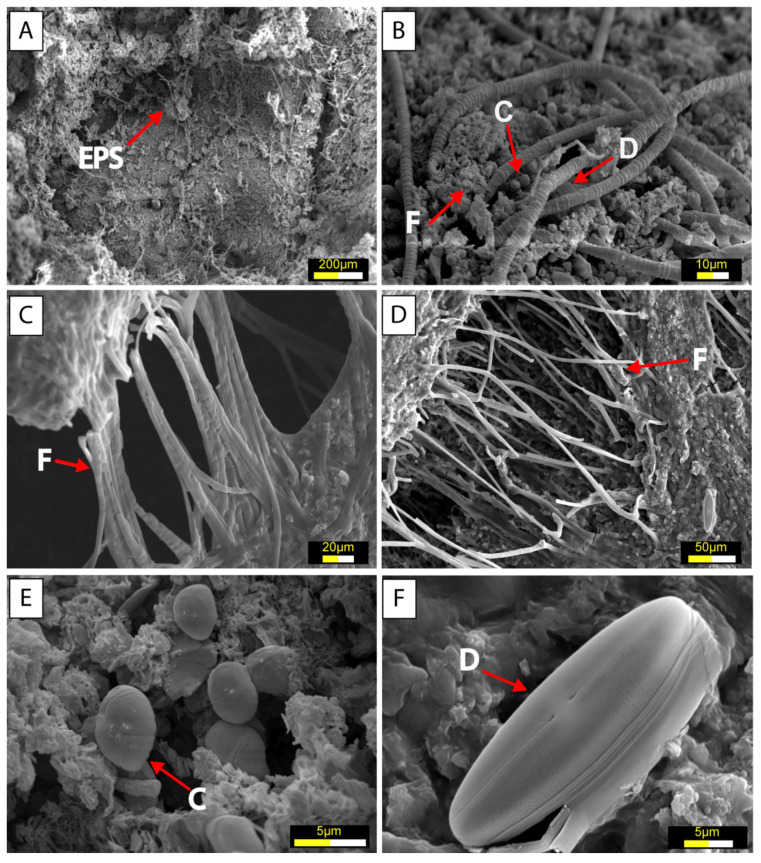
SEM images of the microbial mats samples: (**A**) EPS matrix associated with filamentous cyanobacteria and diatom (TIMO 2); (**B**,**C**) cyanobacteria filaments (TIMO 2); (**D**) filamentous structures interconnected, forming ramifications (TIMO 3); (**E**) coccoid bacteria (TIMO 3); and (**F**) diatom frustules (TIMO 3). Coccoid bacteria (C), Diatoms (D), Filamentous structures (F).

Scanning electron microscopy (SEM) revealed the presence of typical extracellular polymeric substances (EPS) with a cobweb-like appearance. Diatoms, bacteria, and filamentous structures were observed forming thin biofilms within this matrix. These EPS formations were interpreted as products of microbial activity, primarily originating from cyanobacteria and/or diatoms (Figure 7A).

The TIMO 2 sample displayed diverse microbial morphologies, including ramified filaments, coccoid bacteria, and diatoms (Figure 7B,C). Similar textural features were identified in the TIMO 3 sample, which also contained filamentous structures, coccoid bacteria, and diatoms (Figure 7D–F). Among the diatoms, those with larger frustules (Figure 7F) were the least abundant but showed minimal alteration by replacement processes, remaining well preserved.

**Figure 8:**
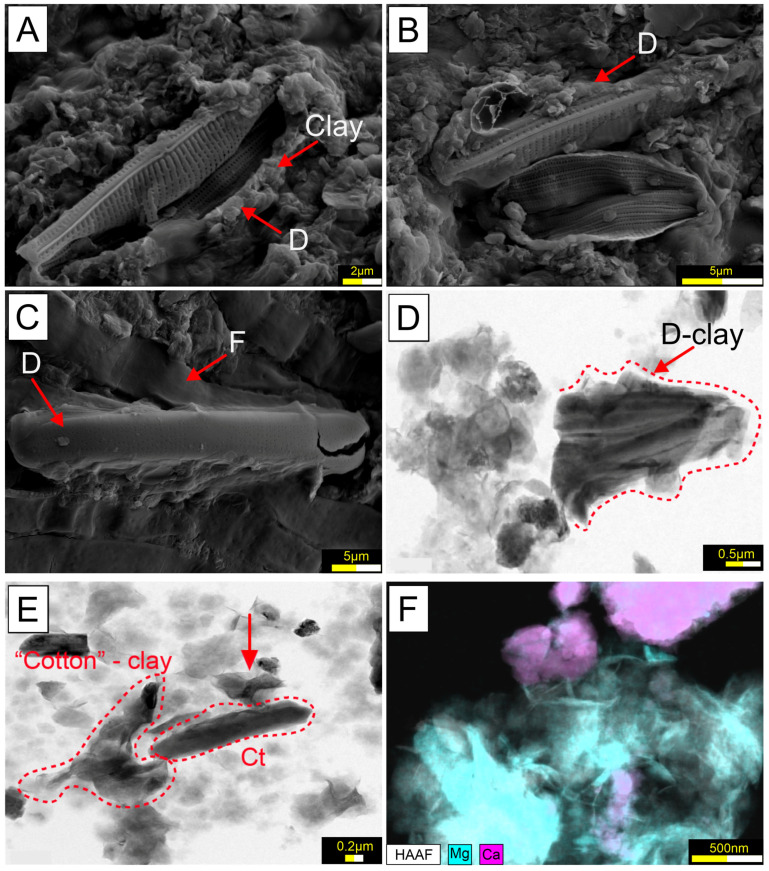
(**A**–**C**) SEM images from TIMO 2 show diatoms’ frustules broken and coated by clay particles; HRTEM images indicate (**D**) the clay coatings on diatom structures and (**E**,**F**) the Mg–clay “coatings” associated with the elongate carbonate crystals (indicated by arrow). Calcite (Ct), Diatoms (D), Filamentous structures (F).

In the TIMO 2 sample, numerous pennate-shaped diatom frustules were identified. These frustules appeared to be partially dissolved and coated with clay minerals (Figure 8A–C). According to [20], the coating corresponds to smectite, a magnesium-rich clay. High-resolution transmission electron microscopy (HRTEM) further revealed that these clay coatings were frequently associated with carbonate mineral phases (Figure 8D–F).

**Figure 9:**
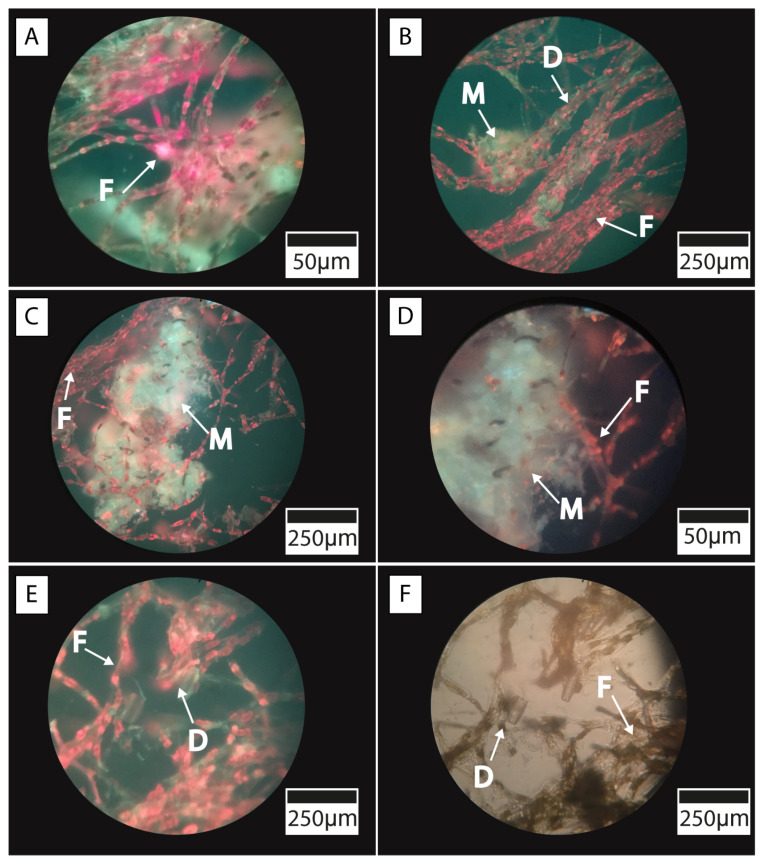
Fluorescence microscope images of microbial mats: (**A**) TIMO 2 sample showing filaments (red fluorescence); (**B**–**D**) filaments, probably from cyanobacteria, associated with mineral aggregates and diatoms (TIMO 2); and (**E**,**F**) TIMO 3 sample also showing a clear relationship between microorganisms and aggregates of calcite. Filaments (F), Mineral aggregates (M), Diatoms (D).

Moreover, fluorescence microscopy, combined with spectral irradiance measurements, revealed distinct absorption regions attributable to the presence of photosynthetic pigments. In the TIMO 2 sample, filamentous microorganisms exhibiting morphological features typical of cyanobacteria were observed. Their presence was confirmed by pronounced scalar irradiance minima at around 680 nm, indicative of chlorophyll absorption, and by the characteristic red autofluorescence emitted by the filaments, which is consistent with the presence of chlorophyll and phycobiliproteins [41] (Figure 9A). In addition, photosynthetic diatom communities and associated crystalline structures were identified (Figure 9B–D). Moreover, minerals appeared to be closely associated with the microbial cells, particularly those of diatoms and cyanobacteria (Figure 9E,F). Filamentous structures were frequently observed encasing calcite grains and forming aggregates with diatoms within the microbial mats.

### 5.3. Chemical Composition of Water, Sediments, and Carbonates

The physicochemical parameters indicate that Laguna Timone is characterized by low temperatures (ca. 4 °C), an alkaline pH (approximately 10), and high salinity, as evidenced by electrical conductivity (EC) values of 120,400 µS/cm and total dissolved solids (TDS) of 147,396 ppm. The water chemistry reveals concentrations of calcium (Ca^2+^) at 141.3 mg/L and magnesium (Mg^2+^) at 448 mg/L. Moreover, the lake exhibits notably high concentrations of sodium (81,000 mg/L), carbonate (54,844 mg/L), and chloride (41,000 mg/L). In addition, characteristic concentrations of potassium (3500 mg/L), nitrate (618 mg/L), sulfate (140 mg/L), phosphates (110 mg/L), and fluoride (12.5 mg/L) were detected (Table 1). The microbial samples developed under these consistent physicochemical conditions, which are maintained by the constant mixing of water along the lake shore and the formation of a wind-induced foam layer.

### 5.4. Isotopic Compositions of Water, Microbial Mats and Carbonates

The microbial mats are spatially associated with carbonate precipitates on the margins of the crater lake. The δ^13^C_DIC_ values associated with the TIMO 2 and TIMO 3 samples show negative δ^13^C_DIC_ values, averaging −13.68‰. The authigenic calcite in the microbial mats displays more positive carbon isotope composition compared with the δ^13^C_DIC_ of the associated water, being higher in the TIMO 3 (δ^13^C 0.8‰VPDB) than the TIMO 2 (δ^13^C −2.8‰VPDB) sample. However, the δ^18^O composition is lower in the TIMO 3 (δ^18^O of −6.5‰VPDB) compared to TIMO 2 (δ^18^O of −4.4‰VPDB). The small-sized carbonate crust formed on top of the TIMO 2 microbial mat shows a similar isotopic composition for δ^13^C of −2.5‰VPDB compared with the mat, but a distinct δ^18^O a value of −7.8‰VPDB (Table 2).

## 6. Discussion

### 6.1. Microbial Diversity of the Microbial Mats and Biofilm of Laguna Timone

In this study, we characterized the composition of autochthonous microbial communities inhabiting a hypersaline crater lake shaped by the distinctive geological and climatic conditions of the sub-Antarctic region of South America. The resulting high alkalinity and salinity make Laguna Timone one of the most extreme environments in the region, one where only alkaliphilic and halotolerant microorganisms can persist.

Hypersaline lakes are known to impose unique constraints on microbial life [45], typically leading to low taxonomic diversity due to the high energetic costs of survival [46]. These conditions require finely tuned nutrient availability and microbial metabolic adaptation. Our findings align with this ecological expectation. Alpha diversity analyses using the Chao1, Simpson, and Shannon indices [47,48] indicate that the microbial communities in Laguna Timone exhibit low taxonomic diversity and are highly specialized, as evidenced by the predominance of a few bacterial and fungal taxa. These patterns reflect the strong influence of extreme environmental conditions, in particular, high salinity and alkalinity. Such factors act as selective pressures that limit the range of organisms capable of survival and favor those with specific physiological adaptations. Furthermore, this highlights the need for further research into the functional roles of these extremophilic organisms. This microbial assemblage reflects a metabolically diverse community dominated by photoautotrophs and complemented by the halotolerant heterotrophs and facultative anaerobes commonly associated with hypersaline microbial mats.

The bacterial genera identified in the microbial mats from Laguna Timone exhibit close phylogenetic relationships with microbial communities from extreme environments worldwide. These environments are characterized by stressors such as high alkalinity and salinity, extreme temperatures, intense UV radiation, and climates ranging from semi-arid to tropical [7,8,9,10,11,12,13,14,15,16,17]. At the phylum level, the dominant bacterial groups identified in the microbial mats, Proteobacteria, Cyanobacteria, and Bacteroidota, are consistent with those reported in other hypersaline ecosystems (e.g., refs. [49,50]). This taxonomic similarity supports the idea that salinity functions as a major environmental filter, potentially exerting a stronger influence than temperature in shaping microbial community composition under these extreme conditions.

At the genus level, TIMO 1 was dominated by taxa known for their tolerance to high alkalinity, salinity, and extreme temperatures [51,52,53,54,55,56,57,58,59,60,61,62]. Notably, *Pseudomonas* has been linked to calcite precipitation in microbialites [55,56,63,64], suggesting a potential geomicrobiological role in carbonate formation within Laguna Timone. Other genera such as *Luteolibacter* and *Flavobacterium*, although present in lower abundance, are also associated with salinity tolerance and may contribute to the functional heterogeneity of this biofilm community [57,58,59,60,61,62,65,66,67].

In TIMO 2, the most abundant genera were *Oscillatoria*, *Indibacter*, and *Nodosilinea*, all of which are commonly reported in hypersaline microbial mats exposed to high pH, thermal stress, and intense UV radiation [17,68,69,70,71,72,73,74,75]. In TIMO 3, the dominant genera included *Belliella* and *Nodularia*, both frequently found in extreme saline environments. *Belliella* is a halotolerant heterotroph known to inhabit coastal salterns and saline lakes [76,77,78,79], while *Nodularia* is a nitrogen-fixing cyanobacterium typically associated with alkaline and hypersaline microbial mats [80,81,82,83,84,85].

These patterns indicate that microbial communities in Laguna Timone are not assembled randomly, but rather reflect strong environmental selection pressures driven by the lake’s extreme physicochemical conditions.

Regarding fungal communities, TIMO 2 was almost exclusively dominated by *Thelebolus* (family Thelebolaceae), a psychrotolerant and halotolerant fungus previously isolated from extreme environments such as the Ross Sea and the Dry Valleys of Antarctica [86,87], where it has also been documented colonizing microbial mats [88]. This extreme dominance may reflect niche specialization under persistent thermal stress. Moreover, *Thelebolus* has been reported from high-latitude soils across Europe and Asia, including glacier fronts in Svalbard and cold regions of Norway and Russia [89,90], suggesting a broad distribution across polar and subpolar biomes.

In TIMO 1 and TIMO 3, fungal genera such as *Plectosphaerella* (Plectosphaerellaceae) and *Ilyonectria* (Nectriaceae) were identified, both commonly associated with plant material and soils. *Plectosphaerella* species are known for their ability to degrade complex organic substrates and have been isolated from saline and low-temperature environments, where they produce hydrolytic enzymes such as chitinases [91,92,93]. Similarly, *Ilyonectria* species, often associated with plants, possess saprotrophic or pathogenic capabilities that enable them to participate in organic matter recycling under challenging environmental conditions [94,95]. Their co-occurrence with other saprotrophic fungi (e.g., *Tetracladium*, *Preussia*) in TIMO 1 suggests possible inputs of plant- or soil-derived materials into the microbial mats. These taxa likely contribute to nutrient cycling within the hypersaline ecosystem by promoting the degradation of organic detritus, even under salinity and low-temperature stress [96].

Taken together, these findings not only expand our understanding of microbial communities in cold hypersaline systems, but also highlight how their structure is shaped by local geochemical conditions and extreme environmental stressors. This work contributes to closing key knowledge gaps in extra-Andean Patagonia and establishes Laguna Timone as a valuable analog for reconstructing early Earth scenarios.

### 6.2. Environmental and Geo-Microbiological Context

The sampling strategy employed in this study effectively captured the spatial variability of microbial activity along the shoreline of Laguna Timone, particularly within the few observable biological outcrops. The environmental conditions of this crater lake, including high solar radiation, exposure to strong winds, salinity, and alkalinity, together with the consequently limited nutrient availability, contribute to the patchy and restricted distribution of microbial activity. As a result, these few localized microhabitats are particularly valuable for understanding microbial diversity and adaptation under extreme environmental stress. One of the most notable observations was the absence of distinct stratification within the microbial mats, which may be attributed to the intense physical reworking driven by wind and wave action. This phenomenon, especially prominent along the eastern shore, likely disrupts mat development, homogenizing the microbial layers and altering their structural complexity.

The close spatial overlap between the microbial mats and marginal sediments composed of basaltic volcanic debris and fluvioglacial input [19] highlights a complex interaction between biological and geological components. These sediments may influence microbial colonization by offering a diversity of textures, porosities, and geochemical substrates. The volcanic sediments, weathered over time, release ions such as Ca, Mg, K, Na, SO_4_^2−^, CO_3_^2−^, Cl^−^, and NO_3_^−^, key elements for microbial metabolism, biosynthesis, and energy generation under hypersaline conditions [97,98,99]. Volcanic sediments may act as a long-term source of chemical nutrients, supporting biological productivity under extreme environmental stress. Previous studies have shown that Quaternary volcanic soils support microbial multifunctionality through the sustained release of carbon, nitrogen, and phosphorus [100]. It is envisaged that basaltic sediment could supply “chemical nutrients” to microbial mats developed under environmental stresses, stimulating biological productivity [101].

On the other hand, the presence of minerals associated to the microbial mats suggests a possible link with the photosynthetic microorganisms. Carbonate aggregates occur, closely attached to the filaments and diatoms. The HRTEM images and EDS analysis show an association consisting of carbonate and clay minerals, and frustules of pennate diatom. The clay mineral was identified by [19] as authigenic smectites. Some studies have documented that an increase in pH driven by oxygenic photosynthesis may also facilitate the dissolution of diatoms composed of amorphous silica; this could explain the elevated levels of dissolved silica within the mats, with the phenomenon further promoting the precipitation of Mg–Si substances [102]. The Mg–clay gel possibly acts as an ideal substrate from which the calcite can precipitate [103,104,105].

In addition, isotopic analyses further illuminate the underlying biogeochemical dynamics. The δ^13^C values measuring dissolved inorganic carbon (DIC) suggest a dominant influence of biogenic CO_2_ derived from microbial respiration and organic matter oxidation [106,107]. Such processes typically deplete ^13^C in DIC pools, especially in heterotroph-dominated systems. In contrast, authigenic carbonates associated with microbial mats display δ^13^C values around −2.6‰. This relative enrichment in ^13^C is consistent with the photosynthetic uptake of CO_2_ by cyanobacteria and diatoms, which preferentially consume ^12^C. As a result, the surrounding DIC pool becomes isotopically heavier, potentially promoting carbonate precipitation. Although there is no clear evidence that microorganisms induce such precipitation or control this system, we cannot rule out the possibility that there exists a relationship between minerals and bacteria. Probably, carbonate values are influenced by evaporative processes, causing fractionation and increases in ^13^C.

These findings underscore the importance of physical and geochemical heterogeneity in shaping microbial community structure in Laguna Timone. The interplay between environmental stressors, such as wind-driven disturbance and nutrient limitation, creates microhabitats in which microbial processes operate under tight energetic constraints. Although the evidence for direct microbial mineralization remains circumstantial, the co-occurrence of specific isotopic signatures and biological structures could be an indication of biological participation in the system. This highlights the potential of crater lakes like Laguna Timone as natural laboratories for exploring microbial resilience and biogeochemical cycling under extreme conditions.

## 7. Conclusions

This study presents the first comprehensive characterization of the microbial communities in Laguna Timone, a hypersaline crater lake in the sub-Antarctic region of South America. By integrating high-throughput sequencing with mineralogical and hydrochemical analyses, we demonstrate that microbial diversity in this extreme environment is low but highly specialized, shaped by strong selective pressures such as salinity, alkalinity, UV exposure, and nutrient scarcity. The identified microbial taxa, both bacterial and fungal, exhibit close phylogenetic affinities with microorganisms inhabiting other cold, arid, and hypersaline environments, such as the Arctic and Nordic regions, and Antarctica. This supports the notion that salinity and other physicochemical constraints act as powerful ecological filters, promoting convergence in microbial community composition across geographically distant but environmentally similar habitats.

Importantly, our results suggest that the development and persistence of microbial communities in Laguna Timone are closely linked to nutrient availability and geochemical dynamics influenced by the volcanic substrate. These findings contribute to closing existing gaps in the geomicrobiological knowledge of extra-Andean Patagonia and align with recent efforts to reconstruct the microbial and environmental history of Quaternary lake systems in the region.

Crater lakes like Laguna Timone provide valuable natural laboratories for exploring microbial adaptation to polyextreme conditions. While not direct analogs of early Earth, they offer meaningful comparative frameworks for investigating microbe–mineral interactions and the formation of potential biosignatures. The data presented here establish a strong baseline for future research using metagenomics, metatranscriptomics, and cultivation-based approaches to further explore microbial functionality, ecological roles, and biogeochemical processes in cold, saline volcanic environments.

## Figures and Tables

**Table 1:** Physicochemical parameters of water samples from Laguna Timone.

Physicochemical Analysis of Water	
Physical Parameters	
Total dissolved solids (mg/L)	147,396
pH	9.7
Electric conductivity (uScm−1)	120,400
Temperature (°C)	4
**Chemistry (mg/L)**	
Calcium (Ca)	141.3
Magnesium (Mg)	448
Sodium (Na)	81,000
Potassium (K)	3500
Iron (Fe)	0.42
Manganese (Mn)	0.25
Chloride (Cl)	41,000
Fluoride (F)	12.5
Sulfates (SO42−)	727.1
Phosphorus (PO3)	110
Nitrates (NH3)	618
Nitrites (NO2)	0.272
Carbonates (CO3)	54,844
Silica (SiO2)	2
Alkalinity Total	65,854

**Table 2:** Mineralogical composition XRD and stable isotope data for the oxygen and carbon of the microbial mats (TIMO 2, 3), carbonates related to the samples of the microbial mats (TIMO 2, 3), and calcite aggregates on the mat from TIMO 2.

Samples	δ^13^C (‰VPDB)	δ^18^O (‰VPDB)	d(A°) Carbonates (DRX)	Mineral Classification (Zhang et al., 2010 [41])
TIMO 2	−2.8	−4.4	3.031	Calcite
TIMO 3	0.8	−6.5	3.031	Calcite
Calcite aggregates (TIMO 2)	−2.5	−7.8	3.031	Calcite

## Data Availability

The "phyloseq" object obtained from the taxonomic analysis excluded sequences with fewer than two reads. Raw sequences were deposited under NCBI SRA BioProject PRJNA1139992.

## Supplementary Materials

The following supporting information can be downloaded at: https://www.mdpi.com/article/10.3390/microorganisms13081957/s1, Figure S1: Relative abundance of bacterial Phylum in TIMO 1 (biofilm), TIMO 2 and TIMO 3 (microbial mats) based on 16S rRNA gene sequencing; Figure S2 Relative abundance of Family in TIMO 1 (biofilm), TIMO 2 and TIMO 3 (microbial mats) based on 16S rRNA gene sequencing; Figure S3: Relative abundance of bacterial Phylum in TIMO 1 (biofilm), TIMO 2 and TIMO 3 (microbial mats) based on 16S rRNA gene sequencing; Figure S4: Relative abundance of Family in TIMO 1 (biofilm), TIMO 2 and TIMO 3 (microbial mats) based on 16S rRNA gene sequencing; Figure S5: Rarefaction curves for bacterial communities based on 16S rRNA gene sequences. Curves display the number of observed ASVs as a function of sequencing depth for samples TIMO 1, TIMO 2, and TIMO 3; Figure S6: Rarefaction curves for fungal communities based on ITS sequences. The number of observed genera is shown as a function of sequencing depth for samples TIMO 1, TIMO 2, and TIMO 3.
