# Supplementary material for: Microbial and Geochemical Diversity of Laguna Timone, an Extreme Hypersaline Crater Lake in Patagonia (52° S)
Source: Microorganisms. 2025 Aug 21;13(8):1957. doi: 10.3390/microorganisms13081957 (PMC12388659; doi:10.3390/microorganisms13081957)
Supplement: Supplementary file 1 [file microorganisms-13-01957-s001.zip › microorganisms-3647975-supplementary.pdf]

Supplementary Material

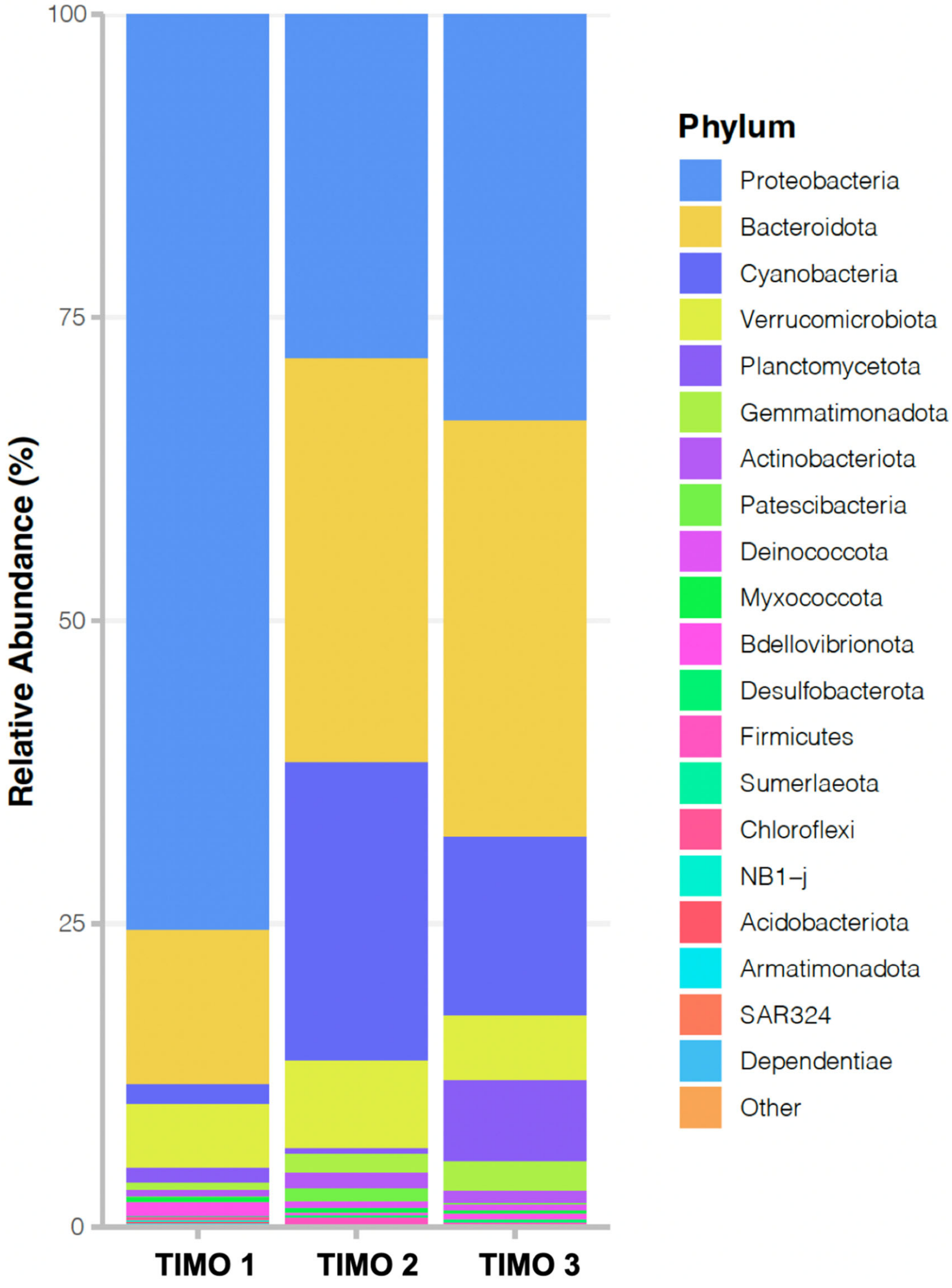

Figure S1. Relative abundance of bacterial Phylum in TIMO 1 (biofilm), TIMO 2 and TIMO 3 (microbial mats) based on 16S rRNA gene sequencing.

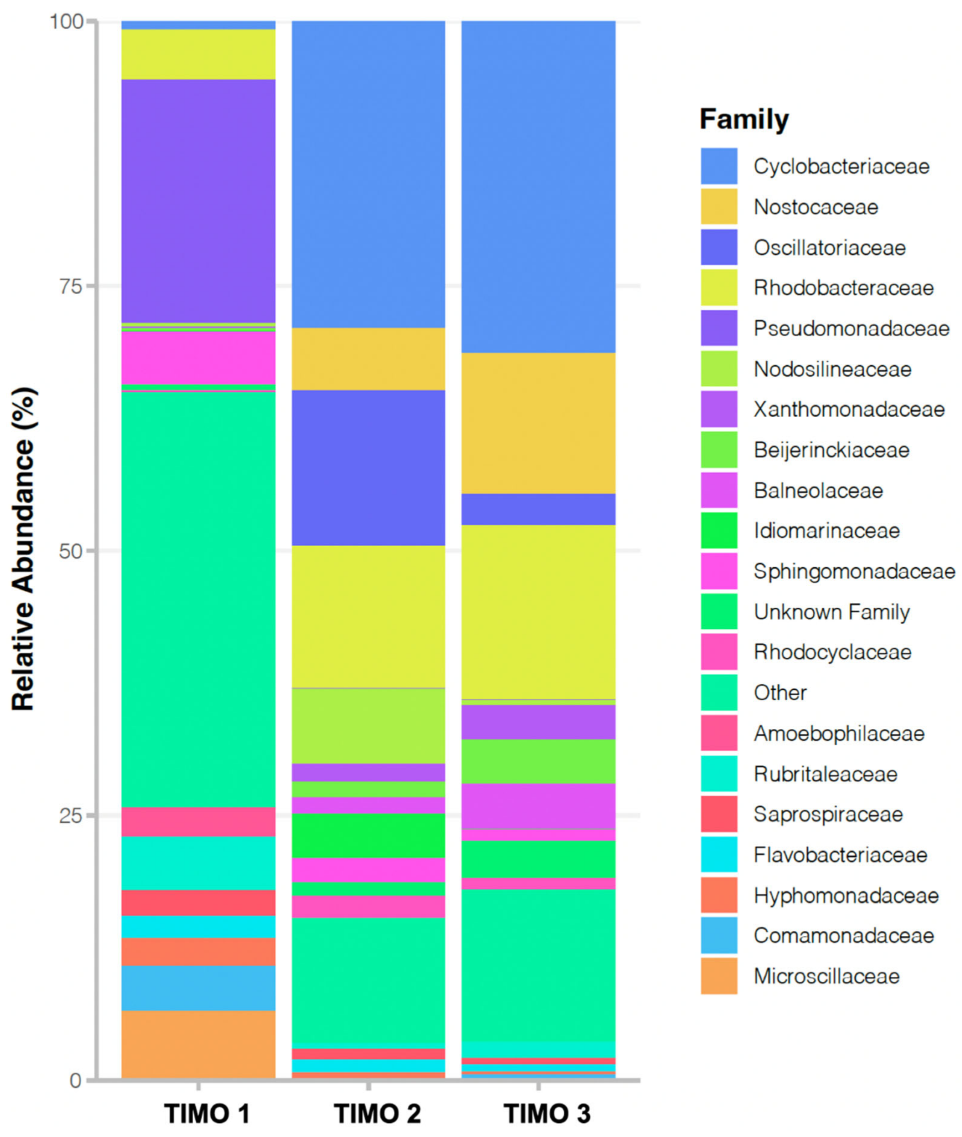

Figure S2. Relative abundance of Family in TIMO 1 (biofilm), TIMO 2 and TIMO 3 (microbial mats) based on 16S rRNA gene sequencing.

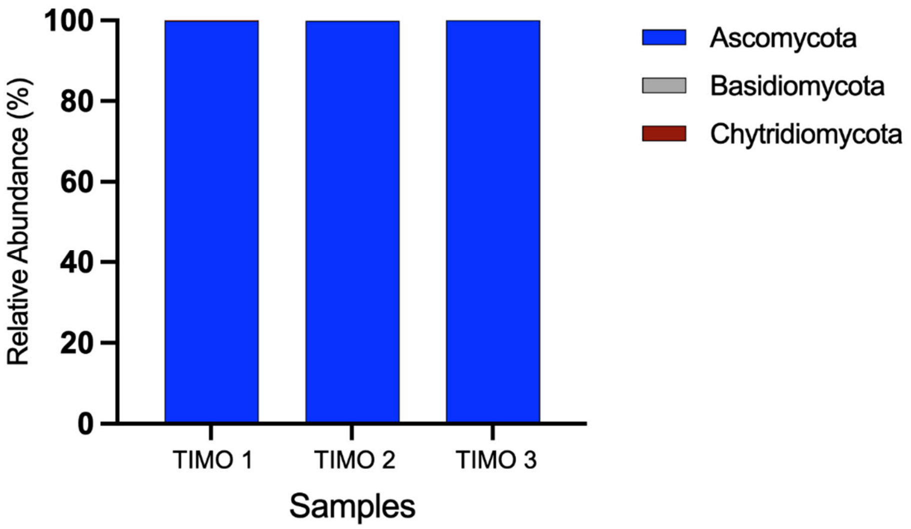

Figure S3. Relative abundance of bacterial Phylum in TIMO 1 (biofilm), TIMO 2 and TIMO 3 (microbial mats) based on 16S rRNA gene sequencing**.**

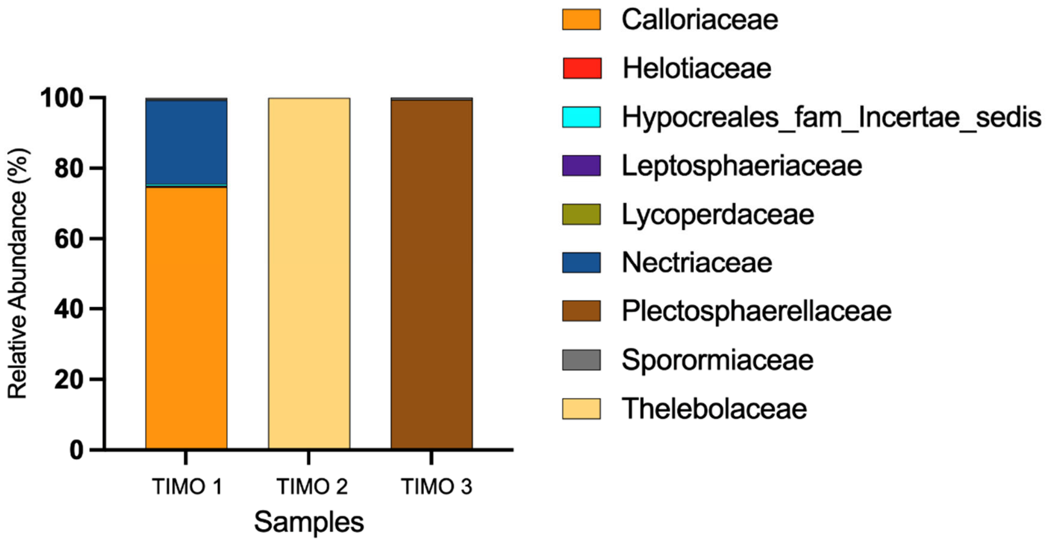

Figure S4. Relative abundance of Family in TIMO 1 (biofilm), TIMO 2 and TIMO 3 (microbial mats) based on 16S rRNA gene sequencing**.**

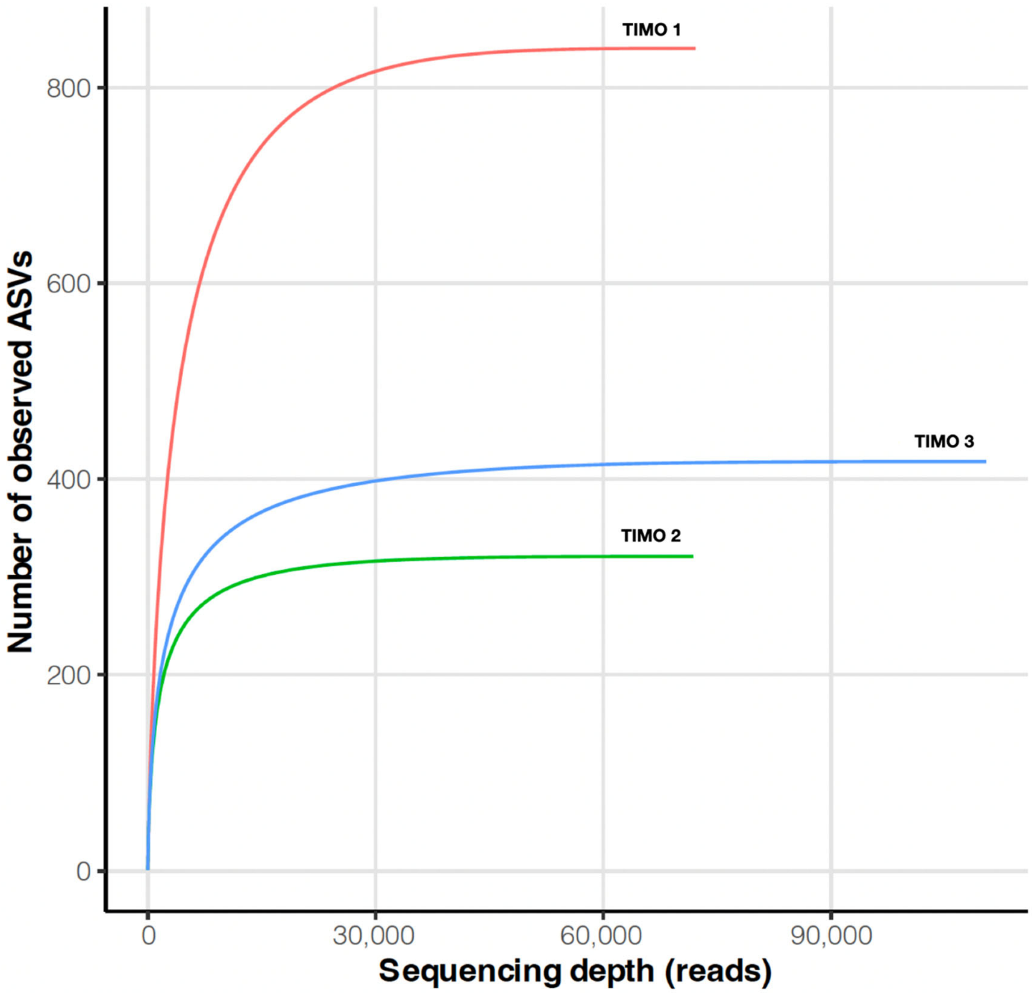

Figure S5. Rarefaction curves for bacterial communities based on 16S rRNA gene sequences. Curves display the number of observed ASVs as a function of sequencing depth for samples TIMO 1, TIMO 2, and TIMO 3.

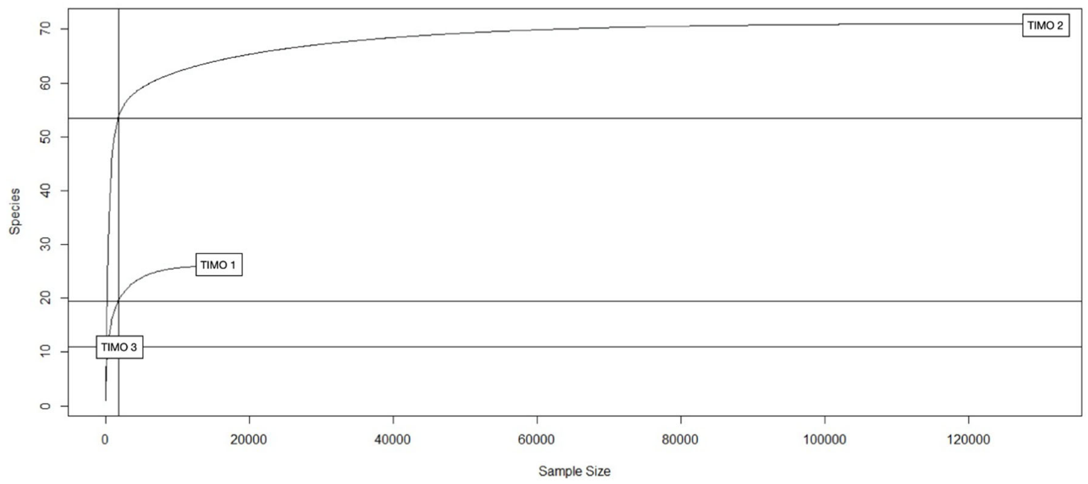

Figure S6. Rarefaction curves for fungal communities based on ITS sequences. The number of observed genera is shown as a function of sequencing depth for samples TIMO 1, TIMO 2, and TIMO 3.
